# Atherosclerotic renal artery stenosis: one year outcome of total and separate kidney function following stenting

**DOI:** 10.1186/1471-2369-5-15

**Published:** 2004-10-15

**Authors:** Giorgio Coen, Eleonora Moscaritolo, Carlo Catalano, Raffaella Lavini, Italo Nofroni, Giuseppe Ronga, Daniela Sardella, Alvaro Zaccaria, Rosario Cianci

**Affiliations:** 1 Renal Pathophysiology and Hypertension Unit, Dept of Medical Sciences, La Sapienza University, Rome, Italy; 2 Dept. of Radiology, La Sapienza University, Rome Italy; 3 6th Medical Clinic, La Sapienza University, Rome, Italy; 4 Dept.Experimental Medicine and Pathology, La Sapienza University, Rome, Italy; 5 Dept. Of Vascular Surgery, La Sapienza University, Rome, Italy

## Abstract

**Background:**

Renal artery stenosis (RAS) is a known cause of hypertension and ischemic nephropathy. Stenting of the artery is a valid approach, in spite of cases of unexpected adverse evolution of renal function.

**Methods:**

In this study, 27 patients with unilateral RAS were subjected to stenting and followed for a period of one year, while 19 patients were observed while on medical treatment only. The group of 27 patients, 67.33 ± 6.8 years of age, creatinine of 2.15 ± 0.9 mg/dl, following stenting, were followed at intervals with biochemical tests, renal scintigraphy and doppler ultrasonography. The control group (70.0 ± 6.1 years, creatinine 1.99 ± 0.7 mg/dl) was also followed for one year.

**Result:**

One year after stenting mean creatinine clearance (Ccr) increased from 36.07 ± 17.2 to 40.4 ± 21.6 ml/min (NS). Arterial BP, decreased after 1,3,6, and 12 months (p < 0.05). The number of antihypertensive drugs also decreased (p < 0.05). A significant increase in proteinuria was also observed. In the control group both Ccr, BP and proteinuria did not show significant changes. Based on renal scintigraphy and Ccr at subsequent times, it was possibile to evaluate the timecourse of renal function in both kidneys of the stented patients. In the stented kidneys Ccr increased significantly. On the controlateral kidney a decrease of renal function (p < 0.05) was observed. Resistance index appeared to be a risk factor of the functional outcome.

**Conclusions:**

Stenting of RAS due to atherosclerosis is followed by stabilization or improvement of Ccr, mainly at the stented kidney, while contralateral renal function showed a decrease.

## Background

Renal artery stenosis due to atherosclerotic changes of the renal arteries has become a serious concern as a cause of hypertension and renal ischemia, resulting frequently in end-stage renal failure [1,2]. Several epidemiologic studies have shown the elevated prevalence of ischemic nephropathy, with special regard to atherosclerotic renal artery stenosis, in elderly patients [3,4]. Instead of the classical surgical approach, percutaneous balloon angioplasty or endovascular stenting have recently become accepted procedures in the attempt to revascularize the stenotic kidney and prevent chronic renal insufficiency. However, in spite of the arterial dilatation obtained with these procedures, there is still some doubt that the long-term outcome is in general satisfactory [5]. There is currently no clear evidence that such interventions prevent further progressive decline of renal function. However the results have been somewhat different in different case series [6,7]. It is known that there are patients with satisfactory results in terms of improvement or stabilization of renal function, while some cases may deteriorate renal function in spite of the dilating procedure [8,9]. As for the results of stenotic artery dilatation procedure on blood pressure, most of reports have confirmed a significant fall in systolic and diastolic blood pressure [10,11], an important finding which however cannot justify the stenting procedure if not accompanied by a consensual improvement in kidney perfusion and stabilization or improvement of renal function. Therefore the purpose of many researchers has been to identify the risk factors which might exclude patients from the revascularizing procedure, due to predictable poor outcome. Radermacher et al. [12] have identified the resistance index (RI) as an important factor predicting the outcome of the stenting. In addition, a limited number of studies [9,13,14] have evaluated not only the overall renal glomerular filtration following the dilating procedure, but also the individual function of the stented and contralateral kidneys. The results are interesting since the behaviour of the two kidneys after the one-sided dilating procedure was found to be divergent. This study provides further data on the evaluation of the two kidneys with a follow-up of one year.

## Methods

The study has been carried out prospectively on 46 patients affected by hemodynamically significant atherosclerotic renal artery stenosis, detected by Magnetic Resonance Angiography or Selective Digital Angiography. All the patients had a unilateral stenosis. 27 patients (diabetes mellitus in 8 cases) were subjected to stenting of the stenotic renal artery while 19 patients (diabetes mellitus in 9 cases) were kept on medical treatment only. Clinical data of the two groups are reported in table 1. Patients were allotted to the control group in case of refusal of the invasive procedure. All patients had a stenosis judged by ultrasonography to be above 70%.

**Table 1:** Clinical and biochemical data of the stented and control groups

	Stented		Control		
**Patient, n°**	27		19		
**age, years**	67,3 ± 6,8		70.0 ± 6,1		n.s
**M/F, n°**	17/10		15/4		n.s.
**Systolic BP, mmHg**	169,1 ± 23		165,8 ± 24,7		n.s.
**Diastolic BP, mmHg**	89,1 ± 14,8		87,4 ± 13,4		n.s.
**Creatinine, mg/dl**	2,15 ± 0,9		1,99 ± 0,7		n.s.
**Cr. Clearance, ml/min**	36,1 ± 17,3		34,6 ± 15,6		n.s.
**Urea, mg/dl**	73,2 ± 36,7		75,5 ± 29,2		n.s.
**Tot. Cholesterol, mg/dl**	236,9 ± 33,8		241,1 ± 41,2		n.s.
**Tryglicerides, mg/dl**	181,2 ± 87,6		166,1 ± 82,8		n.s.
**Sodium, mEq/L**	139,7 ± 5,2		142,0 ± 3,3		n.s.
**Potassium, mEq/L**	4,34 ± 0,5		4,7 ± 0,6		n.s.
**Proteinuria, mg/24 h**	308 ± 323		545 ± 572		n.s.
**Uric acid, mg/dl**	6,8 ± 1,4		6,6 ± 2,3		n.s.
**Resistance Index (DDS)**	0,76± 0,11	(25)	0,79± 0,04		n.s.
**Severity of stenosis (%)**	78,8 ± 8,66	(25)	79,06 ± 9,0		n.s.
		%		%	
**Smoker**	14/27	51,9	12/19	63,2	n.s.
**Hypertension**	25/27	92,6	19/19	100	n.s.
**Diabetes Mellitus**	8/27	29,6	9/19	47,4	n.s.
**Dyslipidemia**	17/27	63	15/19	78,9	n.s.
**Peripheral arterial insuff.**	17/27	63	13/19	76,5	n.s.

In all these patients the renal artery was approached through the femoral artery. French 6 guiding catheter (type "Cobra"or "Bates") was used for selective renal artery angiography and for positioning the stent. All stenotic lesions were repaired with stainless stent Express Vascular SD Monorail 5.5-6-15/20 mm.premounted on a balloon catheter on Choice extra support 014" guide. In these cases primary stenting was performed. The procedure requires, usually, an injection containing 30 ml of 50–50 mixture of isotonic contrast and normal saline.

The patients were followed at the outpatient clinic of the Nephrology unit. Duplex-doppler sonography and renal scintigraphy were carried out basally and following 1,3,6 and 12 months after the stenting procedure. At the same times, biochemical parameters, like serum creatinine and proteinuria were measured. Creatinine clearance was evaluated with the Cockcroft and Gault formula [15].

Doppler ultrasonography was carried out after fasting, following a three days of a low fibre diet and without smoking for a minimum of six hours before the procedure. Patients were studied with an Acuson 120 XP/4 (Acuson Corp., Mountain View, CA), equipped with a 3.5 MHz transducer, with longitudinal anterior, lateral and oblique approach, with at least threefold sampling of parameters along the artery. The standard criteria for the diagnosis of significant renal artery stenosis have been previously reported [4].

Renal radionuclide scintigraphy was performed with a gamma camera (Starcam 4000, General Electric, USA) with 99mTc-DTPA or with 99mTc-MAG3 (mercaptoacetyltriglycine). MAG3 was chosen in patients with creatinine clearance <25 ml/min. Diuretics and/or ACE inhibitors were discontinued at least three days before, if treatment was underway. The criteria of positivity have previously been reported [4].

Evaluation of single kidney renal creatinine clearance was performed by measurement of creatinine clearance and simultaneous renal scintigraphy with MAG3, with percentage-wise function of each kidney, enabling to calculate the creatinine clearance pertaining to each kidney. The entire set of data for this evaluation was available in 21/27 patients.

Statistical analysis. A descriptive univariate analysis, consisting in evaluation of percentages, means and standard deviations has been carried out as first step. To evaluate the dependence among the nominal variables, a Parson's Chi square test was also carried out. In case of not applicable Chi square test due to low theoretical frequencies (<5), Fischer exact test for tables 2 × 2 was employed. Comparison of means of the two groups was made. Since the data did not show a normal distribution, non parametric tests, as Wilcoxon rank-sum test for paired data and Mann-Whitney test, were employed. The significance level was <0.05, as usual. The more interesting significant and not significant data were represented graphically. The data were evaluated with the statistical package BMDP Release 7 (Cork, Ireland,1997).

## Results

Clinical and biochemical data of the treated and control groups are reported in Table 1. There was no significant difference between experimental and control groups.

During 12 months observation period one patient of the stented group began dialysis treatment, while in the control group 4 patients died of cardiovascular events and one patient started the dialysis treatment.

A significant drop in systolic and diastolic blood pressure at all control times compared to basal values, was found in the stented patients, while no significant blood pressure drop was found in the patients not undergoing the PTA stenting procedure (Table 2). A significant fall in number of antihypertensive drugs was also found at 3 and 6 months after the stenting.

**Table 2:** Timecourse of blood pressure (mm Hg), number of antihypertensive drugs and proteinuria (mg/24 h) in the stented and control groups

	**STENTED GROUP**			
	
	**Systolic BP**	**Diastolic BP**	**n° antihypertensive drugs**	**Proteinuria**
**Basal**	169 ± 23	89 ± 14,8	2,07 ± 1,1	309 ± 323
**1 m**	155 ± 12,9*	81,9 ± 6,77*	1,93 ± 1,07	764 ± 690*
**3 m**	157 ± 17,7*	82,0 ± 6,31*	1,6 ± 1,05*	1381 ± 2160
**6 m**	148 ± 12,2*	81,4 ± 5,39*	1,42 ± 0,93*	1743 ± 2884
**12 m**	152 ± 14,4*	81,2 ± 8,87*	1,7 ± 1,17	1377 ± 1643*
	**CONTROL GROUP**			
	
	**Systolic BP**	**Diastolic BP**	**n° antihypertensive drugs**	**Proteinuria**

**Basal**	165,8 ± 24,6	87,4 ± 13,4	2,37 ± 0,83	545 ± 572
**3 m**	163,7 ± 16,5	83,0 ± 10,2	2,26 ± 0,93	534 ± 404
**6 m**	155,6 ± 20,4	88,1 ± 8,45	1,82 ± 0,73	812 ± 536
**12 m**	154,4 ± 20,8	87,1 ± 8,21	2,0 ± 0,94	225 ± 368

(*) = Significant difference compared to basal value (p < 0,05)

A significant increase in proteinuria following the stenting was found at 1 and at 12 months, while the increment in proteinuria observed at the other control times was only borderline significant. There was no diference in the increase of proteinuria between patients with and without diabetes mellitus. No changes in proteinuria was observed in the control group.

**Figure 1:**
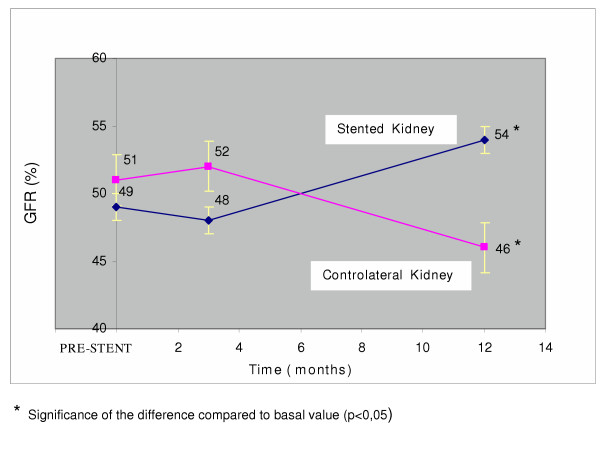
Evolution of percent GFR in the stented and controlateral kidneys.

An increase in creatinine clearance and a slight fall in serum creatinine, however not reaching a significance level, was observed in the stented group, while no increment in creatinine clearance was found in the control group (Table 3). This observation is however limited by the fall in the number of the control group due to death and beginning of dialysis in a total of 5 patients. However the analysis of the separate renal function in the stented and non stented kidneys of the experimental group showed differences in behaviour at the two sides. An increment in the percentage of total glomerular filtration in the stented kidney as a group was found, while a significant fall in percentage of filtration was found on the controlateral side (Fig. 1).

**Table 3:** Evolution of creatinine clearance (ml/min) and serum creatinine (mg/dl).

	**STENTED GROUP**		**CONTROL ROUP**	
	
	**Global Ccr**	**Serum Cr**	**Global Ccr**	**Serum Cr**
**Basal**	36,07 ± 17,2 (27)	2,15 ± 0,94	34,78 ± 15,5 (19)	1,99 ± 0,72
**1 m**	35,29 ± 16,39	2,32 ± 1,33	-	-
**3 m**	34,78 ± 14,38	2,18 ± 0,76	29,99 ± 12,4	2,13 ± 0,73
**6 m**	37,03 ± 18,0	2,15 ± 0,94	33,84 ± 17,9	2,01± 0,66
**12 m**	40,42 ± 21,63 (26)	2,03 ± 0,73	34,78 ± 14,3 (14)	1,98 ± 0,56

**Figure 2:**
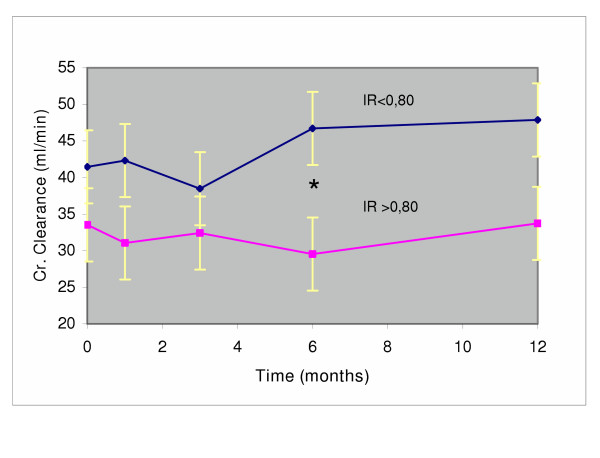
Timecourse of creatinine clearance in stented patients with Resistance index < and > of 0.80. Significance of the difference is indicated (*)

**Figure 3:**
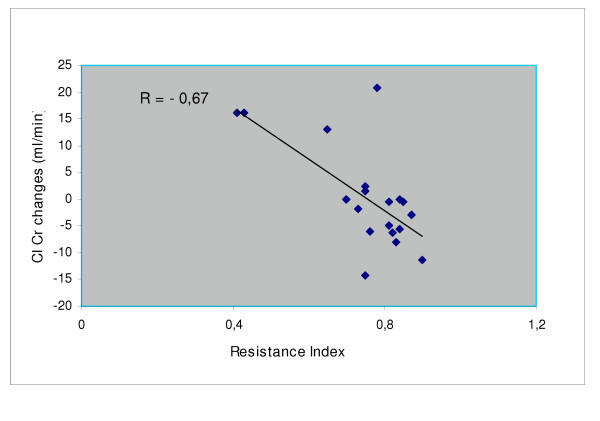
Inverse correlation between Resistance index and changes in creatinine clearance after stenting

In addition, patients with the stent were divided in those cases with a RI above 0.80 and cases with this parameter below 0.80. The patients with lower RI improved, on average, renal function while the patients with elevated RI had a worse outcome (Fig. 2). RI values correlated negatively with changes in creatinine clearance from baselines (r = -0.6712, p < 0.01)(Fig. 3).

## Discussion

The advantage deriving from positioning a stent in a significantly stenotic renal artery has been debated in recent years. Generally favorable results have been reported by Dorros et al [10] on a wide cohort of patients, with special regard to patients with preserved renal function. Lederman et al [8] have found either improvement or stabilization of renal function in 73 % of 300 patients with atherosclerotic renal artery stenosis, bilateral in 48% of cases. Beutler et al. [16] have found similar results on patients with atherosclerotic ostial renal artery stenosis. Perkovi et al [17] consider, as risk factors for an unfavorable outcome, diabetes mellitus, advanced age and renal failure, while the use of ACE inhibitors following the stenting procedure was protective toward death or deterioration of renal failure. Airoldi et al [9] have given a message of caution in extending the dilating procedure to all the patients with renal artery stenosis, due to the low rate of renal improvements and of fall in blood pressure, in their experience, with the finding of at least 20% of restenosis. On the contrary, renal function improvement or stabilization was found in 94% of cases by Rocha-Sing et al. [18], in patients who had a progressive decline of renal function prior to stent implantation.

In our experience, the fall in blood pressure and of the number of antihypertensive drugs was confirmed. Our results on the overall outcome of renal function, over a one year observation period, in patients with one sided renal artery stenosis of atherosclerotic origin, have been satisfactory. As a risk factor of worse outcome, our data have confirmed that RI above 0.80, results in a less satisfactory outcome compared to patients with RI lower than 0.80, as already reported by Radermacher et al. [12]. The stenting procedure, in our experience, was not followed by restenosis or other ischemic complications. Stabilization of renal function observed in the control group should take into account the unfavorable outcome of 5 cases, four deaths and one starting dialysis during the observation period. Anyhow a rational selection of patients who might get benefit from the procedure is advocated. The differences in the percentage of complications following the stenting procedure, as reported in the literature [19], might suggest at least in part the possibility of differences in the individual surgeon's skill in positioning the stent.

As for the increment in proteinuria observed following stenting of renal artery, this finding has not been reported previously, while in basal conditions significant proteinuria in patients with renal artery stenosis has been already reported in the literature [20]. Therefore proteinuria does not exclude the diagnosis of renal ischemia as a cause of renal failure. Proteinuria is probably connected with the type of renal lesions due to chronic ischemia, like focal and segmental glomerulosclerosis, or ischemic glomerular damage. Glomerular lesions resembling focal glomerulosclerosis have been reported in patients with renal artery stenosis [21]. The increase in proteinuria following stenting should probably be attributed to increased perfusion pressure in damaged sclerotic glomeruli.

Less is known of the renal function in the kidney affected by the arterial stenosis, following the stenting procedure, compared to the contralateral kidney. In all our patients renal artery stenosis was of atherosclerotic origin, without cases of fibromuscular dysplasia. In general, no adverse events were found following the stenting in the patients closely followed for one year. In our experience, there were no apparent cases of cholesterol embolization, of thrombosis of the artery, of occlusion of the stent, or of dissecation of the renal artery. The function of the stented kidney improved in most of patients while a reduction of renal function was observed in the controlateral kidney. The overall renal function was stable. Similar findings have been published by Airoldi et al., Leertouwer et al., and La Batide-Alanore et al. [9,13,14]. However their patient cohorts were rather different. One third of the cases of Airoldi et al. [9] were affected by fibromuscular hyperplasia. In only seven of the 27 patients a Palmaz stent was inserted. The increment in glomerular filtration rate of the stenotic kidney was more evident in the cases with fibromuscular dysplasia. Also in the Leertouwer et al. experience [14], renal artery dilatation was carried out in atherosclerotic renal artery stenosis, while the average age of the patients was younger than in our experimental group. Dilatation of the artery was able to induce an improvement of glomerular filtration rate of the treated kidney, although the overall glomerular filtration rate did not change. In La Batide et al. experience [13], 14/32 patients had renal artery stenosis due to fibromuscular hyperplasia and also the average age was decisively younger than our experimental group. Therefore, in our cases, with selective atherosclerotic renal artery disease and an older age, an improvement in function of the stenotic kidney following the stenting procedure was also observed and deserves to be underlined.

The reduction in contralateral kidney function has been attributed to ultrafiltration in the non stenotic kidney, declining after stenting of the stenotic contralateral renal artery. In addition, hemodynamic factors consequent to a decrease in renin-angiotensin activity could be considered as a factor. The involvement of the renin angiotensin system in renal artery stenosis should be suspected due to the significant fall in systolic and diastolic blood pressure following the procedure, an occurrence not found in the control group. Actually, a fall in plasma renin activity or concentration following dilatation of the stenotic artery has been reported by Airoldi et al [9] and by Leertouwer et al. [14].

## Conclusions

In conclusion, the stenting procedure of a stenotic renal artery does not seem to carry important risks, and is accompanied by a definite improvement of the stented kidney, with some reduction of the filtration rate of the controlateral kidney. This event cannot be considered unfavorable, since it denounces a condition of hyperfiltration of the kidney, probably, if left unchanged, able to induce a deterioration of renal function with time. Therefore, also in case of overall stabilization of renal function following the stenting procedure, improvement of the stented side and reduction of hyperfiltration on the contralateral side are both favorable evolutions for long-term success of the revascularization procedure. The results are less satisfactory in patients with RI >0.80. They should probably be excluded from the stenting procedure.

## Competing interests

The author(s) declare that they have no competing interests.

## Authors' contributions

GC, conceived the protocol and cohordinated the study

EM, participated in the design of the study and in the cohordination

CC, was encharged of the magnetic resonance image analysis

RL, collaborated in the duplex doppler ultrasonography examination

IN, was responsible of the statistical examination of data

GR, responsible of renal scintigraphic investigation

DS, performed all the biochemical tests

AZ, performed arteriography and stenting of renal arteries

RC, was active in the ultrasonography investigation and screening of patients with renal artery stenosis

## Pre-publication history

The pre-publication history for this paper can be accessed here:


